# Application of B-Type Natriuretic Peptide in Neonatal Diseases

**DOI:** 10.3389/fped.2021.767173

**Published:** 2021-12-07

**Authors:** Haotai Xie, Yixuan Huo, Qinzheng Chen, Xinlin Hou

**Affiliations:** Department of Neonatal Ward, Peking University First Hospital, Beijing, China

**Keywords:** B-type natriuretic peptide (BNP), neonate, cardiac function, cardiac disease, respiratory disease, congenital heart disease (CHD), patent ductus arteriosus (PDA), pulmonary hypertension (PH)

## Abstract

Numerous congenital or secondary diseases, including, heart disease, respiratory disease, sepsis and many others, can lead to neonatal death. B-type natriuretic peptide (BNP) is a peptide hormone secreted by ventricular cells following an increase in ventricular wall tension. BNP functions to promote vasodilation, diuresis, and sodium release to regulate blood pressure. BNP is a sensitive index reflecting ventricular function, which may aid the diagnosis and monitoring of various neonatal diseases. In neonates, there is currently no consensus on a reference BNP level, as the plasma BNP concentration of healthy newborns varies with age, peaks in the first week after birth, and then gradually decreased to a stable level. In disease states, the correlation between the plasma BNP concentration and the results of echocardiography is good, which is of great significance in the screening, monitoring, and prognosis evaluation of neonatal cardiovascular diseases, including congenital heart disease, patent ductus arteriosus, etcetera. It also facilitates the judgment of the efficacy of treatment and perioperative management. Moreover, the monitoring of plasma BNP concentration provides guidance for the diagnosis, evaluation, and treatment selection of certain neonatal respiratory diseases and neonatal sepsis. This review summarizes the normal BNP values and discusses the application value of BNP in relation to physiological and pathological aspects in neonates.

## Introduction

B-type natriuretic peptide, or B-type natriuretic peptide (BNP), is a member of the natriuretic peptide family in addition to cardiac natriuretic peptide (ANP). BNP is mainly secreted by ventricular myocytes, which respond to the ventricular load, and is widely distributed in the brain, spinal cord, and heart and lung, with the highest content observed in the heart. At present, studies on the pathophysiological significance and clinical diagnostic value of BNP in adults are relatively mature, and BNP can be used as a reliable biomarker for the diagnosis of structural and functional abnormalities of the cardiovascular system. Its application involves the diagnosis, severity evaluation, therapeutic effect evaluation, and prognosis evaluation of various diseases of the cardiovascular system. In recent years, the study of BNP in children has increased; however, due to the particularities of the age of neonates from intrauterine fetal hemodynamics to the neonatal period, the arterial ductus closes, pulmonary artery pressure decreases, and the systemic circulation pressure increases, leading to dynamic changes in early postnatal BNP. In addition, preterm and term infants have different disease spectrums, which are influenced by both developmental factors and diseases. The physiological and pathological significance of BNP is also different between preterm and term infants. This article reviews the normal range of BNP in neonates and the application of BNP in neonatal diseases.

## Manuscript Formatting

### Biological Characteristics and Physiological Functions of BNP

Mammalian cardiac myocytes are composed of a group of phenotypically heterogeneous cells, representing various functions of different subtypes of cardiac myocytes, including electrical pulse generation, electrical pulse conduction, ventricular contraction, atrial contraction, and secretion. The cardiac myocyte contraction-secretion biphenotype is the basis for regulating the secretion of two natriuretic peptide hormones, ANP, and BNP.

BNP is an expression product of the NPPB gene (1), which is mainly synthesized by a p38/NF-κB-dependent mechanism during ventricular wall extension (2). The gene expression process is regulated by inflammatory cytokines, such as IL-1β, tumor necrosis factor, and lipopolysaccharide. This expression process is also upregulated by endothelin and angiotensin (3). NPPB is translated into pre-pro-BNP, further processed as the hormone (pro-BNP) and the final hormone (BNP), and then secreted into the blood circulation. N-terminal proBNP (NT-probNP), also known as a biomarker, is also produced in this process. Pro-BNP processing involves O-glycosylation sites; this is a regulated process that varies over time and is influenced by different pathological states and biosynthesis regions of the heart. Pro-BNP and processed BNP are stored together in specific atrial granules and are processed and co-secreted in response to G-protein coupled receptor signals or following stimulation of secreting cells by atrial muscle traction. At least three mechanisms are involved in the stimulation and secretion of BNP: stretch-activated GQ1α coupling secretion, Gqα coupling secretion, and cytokine-promoting secretion (1). The biological roles of NPs are mediated by receptors, which are membrane-bound guanylate cyclases found in various cells. Three types of NP receptors have been identified: NP receptor type A (NPR-A or NPR1), NPR-B (NPR2), and NPR-C (NPR3). NPR-A is the main receptor for BNP, and BNP binding may lead to changes in the conformation of NPR-A. In the presence of ATP, the inhibition of the protein kinase domain on the guanylate cyclase catalytic site is eliminated, thus increasing the activity of NPR-A guanylate cyclase. BNP binds to NPR-A to induce intracellular cGMP formation, which mediates the relaxation of vascular smooth muscle and skeletal muscle cells through cGMP-dependent protein kinases (PKGs) and cGMP-gated ion channels, leading to vasodilation and muscle relaxation. In addition, cGMP-regulated cyclic nucleotide phosphodiesterase plays various roles in cellular signaling, including regulating cardiac function, adrenal steroid production, and light transduction (1).

All three types of natriuretic peptides (NP) have a common 17-amino acid ring structure that protects the cardiovascular system from volume overload. The storage of BNP is the smallest in atrial granules, where it is synthesized and secreted explosively. The wall stress caused by volume expansion or pressure overload initiates the synthesis of pre-proBNP in ventricular myocytes. BNP also circulates as a hormone in various tissues in the body, promoting vasodilation, and diuresis (4). In addition, BNP also affects the renin-angiotensin-aldosterone system, affects sympathetic nerve activity, inhibits myocardial fibrosis and ventricular remodeling, and has anti-inflammatory effects.

### Normal Value of BNP in Neonates

The plasma BNP level of healthy full-term infants increases immediately after birth. The concentration is relatively high in the first week and then decreases significantly. Subsequently, the BNP level slowly decreases further and gradually reaches a stable level 1 month after birth. The mechanism of the sharp increase in BNP level after birth is not clear, and it is likely to be caused by many factors. The change in the perinatal circulation and the redistribution of blood from the placenta to the lungs increase ventricular volume and pressure load, which stimulates the synthesis of BNP in the left and right atrium and ventricle and secretes it into the circulation after birth (5). However, the level of fetal plasma BNP is higher than that of the placenta, indicating that the placenta has an influence on BNP clearance, which may also explain the increase in neonatal BNP (6). The decrease in plasma BNP can be explained by the fact that the pulmonary circulation pressure gradually increased with age and that diuresis accompanies renal maturation (7).

So far, many groups have reported the reference range of plasma BNP at different times after birth (Table 1). Neves et al. (18) summarized the relevant data, but there are some differences due to differences in gestational age, race, and detection methods. Cantinotti et al. (13) provided detailed BNP data of newborns. Their analysis showed that plasma BNP levels were highest in the first 2 days after birth and gradually decreased in the following days and weeks. Moreover, the BNP levels were not shown to be related to sex and mode of delivery.

**Table 1:** Plasma BNP level of healthy newborns.

**References**	**Age**	***n***	**BNP (pg/mL)**	
			**Median**	**Range**
Koch and Singer (8)	0–1 d	12	231.6	–
	4–6 d	14	48.4	–
Kunii et al. (9)	12–24 h	11	118.8	–
	7 d	11	15.3	–
Soldin et al. (10)	0–31 d	50	–	Percentile: 1,585 (97.5th)
Mannarino et al. (11)	Umbilical cord blood	29	8.6	6.4–13.5
	Day 3	29	59.2	15.6–88.3
	Day 30	29	8.7	5.3–11.7
Cantinotti et al. (12)	0–24 h	57	224	41–837
	25–48 h	49	242	53–866
	49–96 h	50	152	23–862
	97–192 h	32	45	10–739
	8–30 d	34	27	9–63
Cantinotti et al. (13)	0–2 d	66	243.5	Percentile: 468.7 (90th)
	3–4 d	40	169	Percentile: 348.0 (90th)
	5–7 d	20	45	Percentile: 259.5 (90th)
	The first 6 months	33	23	Percentile: 37.2 (90th)
Mannarino et al. (14)	Day 3	34	55.1	23.6–82.7 (IQR)
	Day 28	34	8.9	5.6–20.6 (IQR)
Garofoli et al. (15)	Umbilical cord blood	35	11.4	7.6–15.7 (IQR)
	Day 3	35	55.2	23.6–88.3 (IQR)
	Day 30	35	9	5.8–12.4 (IQR)
Cantinotti et al. (16)	0–2 d	68	243.5	41–866
	3–30 d	75	75	10–741.4
Rodriguez et al. (17)	Umbilical cord blood	146	12.5	7.7–16.8 (IQR)

The BNP level of newborn is related to the growth and development of fetus (Table 2). Mannarino et al. (14) found that the plasma BNP level of preterm newborns without PDA 3 days after birth was lower than that of healthy full-term newborns, but there was no significant difference at 28 days after birth. Tauber et al. (19) studied premature neonates without patent ductus arteriosus and found that the median BNP level was generally lower than the levels identified in the Cantinotti study. Therefore, the BNP level of preterm infants is lower than that of normal full-term newborns in the first few days after birth, although the specific reason remains to be clarified. Torres et al. (20) found that the plasma BNP of monochromic diamniotic twins showed higher levels at birth than did that of singletons. Garofoli et al. (15) found that the umbilical cord BNP concentration of intra uterine growth restriction (IUGR) newborns was higher than that of appropriate for gestational age (AGA) newborns. They also found that the plasma BNP level of IUGR newborns decreased rapidly within 1 month after birth. Therefore, the plasma BNP level of newborns is related to whether they are premature infants, intrauterine growth, twins, or singletons. However, regardless of their growth and development in the fetal period, their BNP levels have a similar trend, indicating that they are affected by the same cardiac circulatory mechanism.

**Table 2:** Plasma BNP level of newborns with different birth conditions.

**References**	**Gestational age (weeks)**	**Neonatal condition**	***n***	**Age**	**BNP (pg/mL)**	
					**Median**	**Range**
Mannarino et al. (14)	25–35	Preterm without PDA^*^	25	Day 3	25.5	10.9–49 (IQR)
				Day 28	15.6	10–22 (IQR)
Garofoli et al. (15)	31–35.6	IUGR^**^	43	Umbilical cord blood	23.9	9.3–68.3 (IQR)
				Day 1	40.6	20.2–105 (IQR)
				Day 3	14	4.9–44.1 (IQR)
				Day 30	11.45	4.9–18.1 (IQR)
Tauber et al. (19)^*^	24–31 6/7	Preterm without PDA	36	Day 1	96	89–213 (95% CI)
				Day 5	16.2	11–30 (95% CI)
				Day 10	10.7	8.9–19.5 (95% CI)
				Day 15	8.3	6.5–13.3(95% CI)
Torres et al. (20)	Mean: 36.3	Uncomplicated MDCA^***^ twin	50	Umbilical cord blood	13.14	9.17–19.84 (IQR)
	Mean: 39.5	Low-risk singleton	27		20.81	16.69–34.01 (IQR)

* *PDA, patent ductus arteriosus*.

** *IUGR, intra uterine growth restriction*.

*** *MDCA, monochorionic diamniotic*.

As there is currently no unified standard for neonatal BNP levels, when explaining the neonatal BNP level, a comprehensive consideration and evaluation should be carried out according to the characteristics of the individual and environment.

### Cardiac Disease

#### Judgment of Cardiac Function

The International Society for Heart and Lung Transplantation defines childhood heart failure as a clinical and pathophysiological syndrome caused by ventricular insufficiency and volume or pressure overload, alone, or in combination (21). The most common cause of heart failure in neonates is structural congenital heart disease (CHD), and the risk and degree of occurrence vary according to the specific type of heart malformation (22). The plasma BNP level reflects the end-diastolic wall stress of the left ventricle in patients with heart failure and can be used for the diagnosis of heart failure (23). The level of serum BNP in neonates is related to the type and severity of CHD, and it has significance for cardiac insufficiency caused by volume load, pressure load, or myocardial disease (24, 25). Therefore, the heart function of newborns can be judged by detecting the level of plasma BNP, and consequently, effective treatment measures can be taken to avoid the occurrence of heart failure and reduce the risk of death and the occurrence of poor prognosis.

##### Cardiac Insufficiency Caused by Volume Overload

Many studies have shown that children with CHD characterized by left ventricular volume overload have higher BNP concentrations, with similar results observed (12, 26, 27) in infants and neonates. Koch et al. (28) found that the BNP level increased in neonates and children with left to right shunt and was positively correlated with shunt flow, right ventricular systolic pressure, mean pulmonary artery pressure, and pulmonary artery resistance, indicating that the plasma BNP level in neonates with congenital heart disease is closely related to cardiac insufficiency caused by volume load. Ventricular septal defect (VSD) is one of the most common congenital heart malformations. The volume load of left atrium and ventricle increases in patients with large ventricular septal defect without pulmonary vascular disease (29). Some studies have shown that plasma BNP levels are elevated in children with VSD and are significantly correlated with the ratio of pulmonary blood flow/systemic blood flow (QP/QS) and left and right ventricular end diastolic volumes (30–32). For newborns, the study of Kunii et al. also has similar findings as children (9). Davlouros et al. (33) also found that plasma BNP levels were significantly higher in volume loaded Left to Right (LtR) shunt newborns than in neonates with haemodynamically insignificant LtR shunts. Therefore, plasma BNP level may reflect the degree of ventricular dysfunction caused by neonatal volume load.

##### Cardiac Insufficiency Caused by Pressure Overload

Pressure overload, also known as afterload overload, is an increase in the resistance load that the heart bears during contraction; it includes left ventricular pressure overload and right ventricular pressure overload. For neonates, left ventricular pressure overload is mainly caused by obstructive CHD on the left side of left ventricular pressure, including aortic stenosis, coarctation of aorta, and Shone complex. Pressure overload near the stenosis (left ventricle) leads to increased systolic wall tension and hypertrophy, decreased ventricular compliance, and increased left ventricular end-diastolic and left atrial pressure, resulting in cardiac insufficiency (34). Cowley et al. (26) studied 96 children with different types of CHD and found that left ventricular outflow tract obstruction and left ventricular pressure load were related to BNP levels and that the BNP level increased as the degree of obstruction increased. Das et al. (35) studied 122 CHD neonates with left obstructive lesions and found that the BNP level of the neonates was elevated, especially in neonates with critical congenital heart disease, whose BNP ranged from 553 to > 5,000 pg/ml. A significant increase in plasma BNP levels was also observed in newborns withcoarctation of aorta (36). Therefore, the BNP level is a good indicator for neonatal heart insufficiency caused by pressure load, especially for left obstructive lesions.

##### Cardiac Insufficiency Due to Myocardium

*Cardiomyopathy* The change in BNP can predict the cardiac dysfunction caused by various etiologies. Hayakawa et al. (37) found that 23.5% of children treated with Adriamycin had left ventricular dysfunction. Moreover, compared to the normal control group (*P* < 0.01) and the normal heart function group (*P* < 0.05), the plasma BNP level of patients treated with Adriamycin was significantly increased. Notably, BNP levels were significantly associated with systolic function, not diastolic function. It has also been demonstrated that plasma BNP level may be a marker of doxorubicin-induced cardiac toxicity in children. As a quantitative biomarker of heart failure, BNP is closely related to the stimulation of ventricular myocytes by transmural pressure. Therefore, the classification of cardiomyopathy according to morphology may be of more significance to the study of BNP.

BNP plays a diagnostic role in some types of cardiomyopathy but not in others. Restrictive cardiomyopathy (RCM) is characterized by increased ventricular wall stiffness, decreased diastolic function, and limited filling. In the early stage, the atrium is significantly dilated, and the systolic function of the left ventricle is significantly decreased with the progression of disease. BNP levels have been shown to be significantly higher in neonates with RCM. BNP can also be used to distinguish RCM from constrictive pericarditis as BNP is higher in patients with RCM than in those with constrictive pericarditis. Although there may be some overlap, a clearer distinction can be made by also considering the tissue Doppler phenomenon (38). Hypertrophic cardiomyopathy (HCM) is a hereditary cardiomyopathy characterized by asymmetrical hypertrophy of the ventricular septum. Öner et al. (39) demonstrated that the sensitivity of the BNP level mainly reflects the degree of left ventricular hypertrophy and has no obvious correlation with the presence or absence of obstruction of left ventricular outflow tract in patients with HCM. Therefore, it cannot be used to identify the types of HCM. In addition, cases of significantly elevated BNP in neonatal enterovirus myocarditis have been reported. These results indicate that BNP may have some diagnostic value for infectious myocarditis (40).

However, BNP has prognostic value in some types of cardiomyopathy. Dilated cardiomyopathy (DCM) is characterized by the enlargement of the left or both ventricles with systolic dysfunction and often leads to ventricular arrhythmias, heart failure, and death. The study of Noori et al. (41) demonstrated that the BNP level in patients with DCM was significantly correlated with multiple echocardiographic results, reflecting overload damage of ventricular function. Moreover, the severity of the disease in Ross grade was significantly correlated with the BNP level. Some studies have shown that BNP has high sensitivity in the clinical prediction of HCM. Indeed, Öner et al. (39) demonstrated that BNP levels higher than 98 pg/mL, a z-value of septal thickness > 6, and a high mitral and ventricular septal E/EA ratio may be used to indicate life-threatening conditions in patients with HCM.

*Severity of Neonatal Asphyxia Based on the Degree of Myocardial Injury After Hypoxia*. Neonatal asphyxia is a common emergency after birth and is an important factor of neonatal disability and death. Early diagnosis of neonatal asphyxia is important to take the correct measures to improve the survival rate and reduce the incidence of complications (42).

Neonatal asphyxia can cause organ ischemia and hypoxic injury, mainly accumulated in the heart and brain. Studies show that myocardial damage caused by asphyxia can occur in 28–73% of neonatal asphyxia cases, which is the main cause of neonatal asphyxia death. CTnI, cTnT, and CK-MB are myocardial injury markers, the elevation of which can be seen in patients with neonatal asphyxia; however, they are susceptible to confounding factors, such as gestational age and prenatal hormone use. Therefore, their use in the diagnosis of neonatal asphyxia is limited. BNP is a marker reflecting the structure and function of the heart, with high sensitivity and specificity, and is suitable for the diagnosis and severity assessment of neonatal asphyxia (42).

Cetin et al. (43) highlighted that the plasma BNP concentration in children with perinatal asphyxia on the first postpartum day was significantly higher than that in the control group, suggesting that BNP could be used as an indicator for the diagnosis of neonatal asphyxia. Jiang et al. (42) drew similar conclusions, showing that the plasma BNP concentration at 12 h postpartum in the asphyxia group was significantly higher than that in the control group without asphyxia and myocardial injury. The study of Vijlbrief et al. showed that when neonates with perinatal asphyxia received hypothermia treatment, their level of BNP was lower than that of the group who didn't. This indicates that BNP level may indicates the effect of asphyxia treatment (44). Plasma BNP is a rapid and simple laboratory indicator for the diagnosis, severity, and prognosis of neonatal asphyxia. Timely clinical examination of BNP in children with suspected asphyxia is helpful for early diagnosis and treatment of asphyxia, which is of great significance to reduce myocardial damage caused by ischemia and hypoxia and to reduce the incidence and mortality of sequelae.

#### Cardiac Function Management

##### Perioperative Management of Cardiac Function

BNP is generally higher after neonatal heart surgery than before, which is a general pattern associated with perioperative changes. However, variations in BNP levels that defy this pattern may have different meanings. The perioperative characteristics of BNP may be different from those of adult patients due to unstable baseline BNP in healthy newborns and other factors such as BNP metabolism. In general, preoperative biomarker levels are relatively easier to interpret clinically than post-operative levels, which may be influenced by a number of confounding factors, such as patient age (day of age) and disease severity. In older children, postoperative biomarker levels are typically higher than preoperative levels, with levels peaking 12–24 h after surgery and remaining higher than preoperative levels at discharge. However, Cantinotti et al. (16) suggested that, on average, neonates showed very high preoperative BNP levels and a progressive decline in postoperative biomarker levels. This different postoperative BNP dynamic pattern may be due, in part, to the generally higher severity of neonatal congenital heart disease and the greater complexity of neonatal surgery. From this perspective, an early postoperative significant no decline or gradual increase in neonatal BNP levels may indicate complications, and/or neonatal CHD may only be partially resolved surgically. Even so, newborns are often discharged from hospital with higher levels of BNP than healthy babies of the same age (16). Thus, even in patients with well-corrected heart defects, hemodynamic homeostatic restoration is slow.

BNP plays a role in preoperative and intraoperative evaluation of patients with heart failure and cardiac interventional surgery. Pichette et al. (45) included multiple biomarkers such as BNP in the preoperative evaluation of patients with heart failure or at risk of heart failure but also suggested that goals should be individualized, with an individualized target threshold and consideration of delayed surgery in patients with rapidly rising BNP levels. However, in the neonatal context, there is no mention of how to distinguish between a physiological increase in BNP and a pathological increase in BNP. However, several studies have assessed the role of BNP in children receiving mid-term mechanical cardiac support. These studies show that BNP is accurate in predicting and detecting rejection and that biomarker levels decline after cardiac mechanical support. However, further studies are needed to better demonstrate the role of BNP testing in these specific clinical settings (16).

In neonates undergoing cardiac surgery, BNP levels are predictive of short-term outcomes in the hospital. In the BNP composite score designed by Niedner et al. (46), the mean BNP 24 h after surgery had the highest overall correlation with postoperative outcome and was, therefore, the preferred BNP index for further analysis. Both peak postoperative BNP and 24-h mean BNP were significantly associated with each type of postoperative outcome. The most significant of which were thoracotomy time, ventilation time, length of intensive care unit (ICU) stay, and length of hospital stay (*r* = 0.51–0.65, all *P* < 0.001). Several studies have shown that the higher the level of BNP in pediatric cardiac surgery patients before surgery, the worse the prognosis, the longer the time of intubation, and the longer the ICU stay. In a linear regression analysis of 336 pediatric cardiac surgery patients, Cantinotti et al. (47) found a correlation between increased preoperative BNP and intubation time (*P* < 0.001). In multivariate logic analysis, an independent correlation between preoperative BNP and intubation time was shown in 89 newborns (*P* = 0.039). Kim et al. (48) also showed an independent correlation between preoperative BNP and intubation time in patients with transposition of the great arteries through linear multivariate analysis (*P* = 0.005). The study by Nahum et al. (49) showed that increased BNP before surgery was significantly associated with prolonged ICU stay (*P* = 0.05) (50). The results of a study by Kanazawa et al. showed a close relationship between preoperative BNP and prolonged ICU stay. Kanazawa et al. also showed that preoperative neonatal BNP was independently associated with postoperative and postoperative adverse events, including (1) death in the ICU, (2) need for extracorporeal membrane oxygenation, (3) cardiac arrest, and (4) hemodynamic instability requiring reoperation.

BNP level may be related to postoperative development in newborns. In the study by Butts et al. (51), multivariate longitudinal analysis of neonatal single-ventricular echocardiography measurements found that higher logBNP was associated with greater end-systolic volume Z-score and degree of atrioventricular regurgitate. BNP was also associated with postoperative body length, body weight, and mental and motor development. Therefore, an abnormally high BNP level may indicate that the development of the newborn after surgery is affected.

In general, BNP has a certain predictive value in the operation of neonatal heart-related diseases, but how to eliminate confounding factors to achieve the application effect of clinical practice remains to be explored. The predictive effect of BNP on prognosis in non-cardiac surgery is supported by some studies in adults, but such data are lacking in neonates.

##### Management of Cardiac Function in Sepsis

Neonatal sepsis can be serious, from subclinical infection to focal or systemic disease, with complications including pulmonary hypertension, heart failure, and other cardiovascular system symptoms, resulting in septic shock and even death of the newborn (52). BNP has been found to guide the diagnosis and prognosis of cardiac insufficiency in sepsis (53, 54).

Circulatory dysfunction in children with septic shock is associated with mortality and is an independent risk factor for death in children with septic shock (55, 56). Williams et al. (57) found that myocardial dysfunction was prevalent in patients with pediatric septic shock (PSS) and that left ventricular systolic dysfunction was associated with higher disease severity, vasoactive use, and BNP level. However, the significance of these optimal bounds for neonatal sepsis is limited due to the large variation of plasma BNP levels in neonates after birth.

#### Application of Patent Ductus Arteriosus (PDA)

##### Diagnosis in Hemodynamically Significant PDA

In full-term infants, the ductus arteriosus usually contracts after birth and functionally closes within 72 h. However, in premature infants, the closure is delayed, which may lead to PDA. Some children will present a series of clinical symptoms and complications, some of which may even be life-threatening, known as symptomatic PDA (sPDA). Significant hemodynamics is often used to distinguish between meaningful and insignificant patent ductus arteriosus. Therefore, early diagnosis of hemodynamically significant patent ductus arteriosus (hsPDA) is of great significance (58). In recent years, studies have found that BNP levels have clinical application value for evaluating PDA (59).

At present, there is no standard best cutoff value for the diagnosis of hsPDA or sPDA using BNP level, but some studies have shown that the predictive diagnosis of PDA based on blood plasma BNP levels in newborns within 7 days after birth has value (Table 3). Tauber et al. (19) found that BNP levels in preterm infants without PDA increased significantly on the first day after birth, decreased on the fifth day, and remained at a low level thereafter, while gestational age had no significant effect on BNP levels; this study helped to increase the understanding of BNP levels in preterm infants with PDA. Moreover, Jeong et al. (71) found that the BNP level of premature infants with PDA was correlated with many parameters of echocardiography and that the BNP level on the second day was positively correlated with the left atrial diameter/aortic diameter, which had guiding significance for the diagnosis of PDA. Furthermore, a systematic review of ten BNP studies (72) showed that the accuracy of BNP level in the diagnosis of PDA varies greatly due to different detection methods, gestational age, and the age of children. Therefore, it is recommended to verify the BNP detection for specific patient populations to determine the treatment plan (Table 3).

**Table 3:** Plasma BNP level in the diagnosis of PDA in neonates.

**References**	***n***	**Gestational age (weeks)**	**Age**	**BNP cutoff value (ng/L)**	**Sensitivity (%)**	**Specificity (%)**
Sanjeev et al. (60)	14	24–31	2–28 d	70	92.9	73.3
Choi et al. (61)	66	25–34	3 d	1,110	100	95.3
Flynn et al. (62)	20	24–35	4–5 d	300	52	100
Czernik et al. (63)	67	<28	2 d	550	83	86
Kalra et al. (64)	52	<34	3–7 d	122.5	100	100
Kim and Shim (65)	28	<37	4 d	412	100	95
Lee et al. (66)	73	Median 27.1	24 h	200	83.9	61.9
Lee et al. (66)	73	Median 27.1	24 h	900	54.8	95.2
Mine et al. (67)	46	<33	24–48 h	550	83	86
Gao et al. (68)	63	<34	2–3 d	292.5	75.4	77.6
Lee et al. (69)	63	<35	3 d	722	–	–
Parra-Bravo et al. (70)	29	<32	3–5 d	486.5	81	92

##### Determination of PDA Severity and Treatment of PDA

PDA is a relatively common disease in CHD. At present, many studies have shown that there is a significant correlation between the BNP level of children and the size of PDA (64, 73, 74). König et al. (73) studied 46 neonates with PDA and found that the average diameter of the PDA was 3.2 mm and the BNP level of the neonates was significantly correlated with the size of the PDA. Some studies have also found that a variety of indicators are related to the persistence of PDA, including high-level BNP (75). Elsayed et al. (76) found that BNP level in premature infants with hsPDA was negatively correlated with hemodynamic parameters of whole body blood flow at 48–72 h and was positively correlated with the pulsatility and resistance index of the middle cerebral artery and coronary artery, which reflected the physiological predictive value of BNP before symptoms of PDA. Moreover, Elsayed et al. (74) found that the BNP levels increased significantly with the increase of PDA diameter for neonates with PDA aged from 2 to 3 d. Meanwhile, the number of days of oxygen inhalation and the number of PDA drug treatments or surgical ligations also increased significantly. The BNP cutoff value for predicting any adverse prognosis in children with PDA was 90 pg/ml, with a sensitivity of 71% and a specificity of 95%. Therefore, the BNP level has a significant correlation with the diameter and persistence of PDA, which can reflect the severity and prognosis of neonates with PDA, and facilitates treatment selection for neonates with PDA.

The plasma BNP level of PDA neonates is significant for treatment decision-making. Non-steroidal anti-inflammatory drugs and other drugs are used to close PDA to prevent such complications. Indomethacin and ibuprofen are considered the standard drugs for the treatment of PDA (77). Hsu et al. (78) used indomethacin to treat 31 preterm neonates (mean gestational age 30 weeks) with sPDA and found that the cutoff value of BNP for predicting the unresponsiveness of premature neonates with sPDA to indomethacin treatment was 1,805 pg/ml, with a sensitivity of 88% and a specificity of 87%. A high baseline BNP level indicates that premature neonates with PDA have a poor response to indomethacin and need surgical treatment. Mine et al. (67) found that a BNP level of 250 pg/ml at 24–48 h after birth was the best cutoff value for predicting indomethacin treatment in premature neonates with hsPDA, and the maximum BNP level of 2,000 pg/ml at 5 days before birth was the best cutoff value for predicting surgical ligation. Oh et al. (79) studied 92 cases of premature neonates with hsPDA treated with ibuprofen in the first course (IBU1) and 19 cases in the second course (IBU2) and found that the cutoff values of effective BNP levels were 331 pg/ml (sensitivity 75.0%, specificity 80.9%) and 423 pg/ml (sensitivity 78.6%, specificity 100%), respectively. However, no cutoff value of baseline BNP level was established to predict the efficacy of ibuprofen in premature neonates with hsPDA. Shin et al. (80) found that ibuprofen can be stopped when the estimated BNP level is lower than 600 pg/mL to avoid unnecessary cyclooxygenase inhibitor doses and that this does not increase catheter closure failure and short-term morbidity related to sPDA. Moreover, King et al. (81) used the BNP level to assess the use of acetaminophen in infants with hsPDA older than 2 weeks, and the BNP level of some infants with hsPDA decreased after treatment. Therefore, the BNP level can be used to predict whether a newborn with PDA needs medication or surgery, and it provides guidance regarding the efficacy of the drug and the time of drug withdrawal.

#### Diagnosis of CHD

CHD has a very high mortality rate worldwide, and its prevalence is gradually increasing (82). Moreover, CHD is the most common cause of heart failure in children. However, routine examinations of some newborns with CHD may be normal, and the signs of critical CHD may not be obvious until after hospital discharge (83). Nearly 60% of HF cases in pediatric patients occur within the first year of life (84). Therefore, early detection and diagnosis of CHD are of great significance to the prognosis of children. Cardiac biomarkers such as BNP are simple and effective tools for assessing CHD, and they also play a role in determining the diagnosis and treatment of pediatric heart disease patients (85).

Some studies have shown that BNP plays a role in the diagnosis of CHD in children and adults and can help identify critical heart disease in children in acute care settings (86, 87). For newborns, BNP can be used as an auxiliary marker for the diagnosis of CHD and can also be used to perform a comprehensive evaluation of children to help determine the severity and progress of heart failure and monitor the treatment response (24) (Table 4).

**Table 4:** BNP in the diagnoses of CHD.

**References**	**Disease**	***n***	**Age**	**BNP cutoff value (pg/ml)**	**Sensitivity (%)**	**Specificity (%)**
Law et al. (88)	Cardiovascular diseases	42	0–7 d	170	94	73
Cantinotti et al. (89)	CHD	152	0–3 d	417	84	66
			4–30 d	206	91	80
Cantinotti et al. (90)	CHD	218	0–4 d	363.5	78	87
			5–30 d	109.5	94	92
Davlouros et al. (33)	hsLtR^*^ CHD	75	12 h or 2 d	132.5	93.1	100

* *hsLtR, hemodynamically significant Left to Right shunts*.

It has been shown that BNP levels are significantly increased in neonates with cardiovascular disease (88). The age of newborns after birth plays an important role in the best cutoff value of plasma BNP in the diagnosis of neonatal CHD (89, 90). At present, there is no standard best cutoff value for the diagnosis of neonatal CHD using BNP level, but some studies have shown that the predictive diagnosis of CHD in the blood plasma BNP level of newborns has clinical value (Table 4). However, despite using the same kit for research, the results of Cantinotti and Davlouros were somewhat different. This may be because the neonatal plasma BNP level changed significantly within the 4 days after birth. Furthermore, the platform used to determine BNP was different, the number of children included in the sample was different, and the diagnostic criteria were also different. Therefore, clinicians should pay special attention to the age of newborns during BNP detection and the kits and detection platforms used in order to improve the accuracy of the diagnosis of children with CHD.

There is also a certain relationship between the severity of neonatal CHD and the level of plasma BNP. Das et al. (35) showed that BNP levels were elevated in all critically ill neonates with congenital heart disease with shock, and reported the best cut BNP level to predict shock in patients with CHD was 490 pg/ml, with a sensitivity of 100%, a specificity of 41%, and an accuracy of 58%. Cantinotti et al. (90) also showed elevated BNP levels in newborns with complex coronary heart disease. Davlouros et al. (33) found that plasma BNP levels were associated with the severity of volume load in newborns with LtR shunt. However, in the first few hours of life, when rapid physiological changes occur in cardiac hemodynamics, it may be difficult to understand the severity of the disease (25). At present, the available data on BNP level and CHD severity are limited, and further research is needed to better clarify the role of neonatal BNP level in assessing the severity of CHD.

Plasma BNP can also be combined with other detection indicators to predict neonatal CHD. Sahin-Uysal et al. (91) attempted to use maternal blood and cord blood BNP to predict neonatal CHD. However, the results showed that elevated cord blood BNP levels could be used as a predictor of neonatal congenital heart disease and an indicator of poor prognosis, while BNP levels in maternal blood had no relevant significance. Dasgupta et al. (92) found that the positive predictive rate of cardiac hypertrophy in neonates using chest X-ray alone to predict heart disease was only 15%. If combined with abnormal echocardiography or BNP > 100 pg/ml, the positive predictive value would be greatly increased. Therefore, in the absence of echocardiography, in addition to the detection of neonatal plasma BNP levels, the combined use of chest X-rays, electrocardiograms, and other laboratory tests can better predict the occurrence of neonatal CHD.

### Respiratory Diseases

#### Application of BNP in Persistent Pulmonary Hypertension (PPHN)

##### PH and Persistent Pulmonary Hypertension in Newborns (PPHN)

PH is a pathophysiological state of elevated pulmonary artery pressure in the resting state (right heart catheter ≥ 25 mmHg at sea level), which can be accompanied by different degrees of right heart dysfunction (93). Due to the change from fetal blood circulation to postnatal blood circulation, pulmonary vascular resistance is continuously increased after birth, resulting in the shunting of blood from the right heart to the left heart in the atrium or ductus arteriosus, known as persistent pulmonary hypertension in newborns (PPHN) (94). The clinical manifestations of PPHN are severe hypoxemia and respiratory failure, which can be complicated with cardiac insufficiency, multiple organ failure, and disseminated intravascular coagulation. The incidence rate of PPHN is ~1.2–4.6/1,000 live births, and the mortality rate is ~10–20%. Some patients even suffer chronic lung disease and neurodevelopmental sequelae after rehabilitation (95). Early diagnosis and appropriate treatment are of great significance to improve the prognosis and reduce mortality.

##### Application of BNP in PPHN

For infants without congenital heart disease, BNP is a simple and non-interference measurement index to evaluate PPHN. The study of Fu and Zhang (96) found that the BNP had good correlation with the echocardiography index (recent “gold standard”), such as right atrial pressure (RA), right ventricular pressure (RV), and tricuspid regurgitation pressure gradient (TRPG). Compared to echocardiography, BNP is simpler and more widely available, so it can be used as an alternative index of echocardiography for PPHN screening. Reynolds et al. (97) found that BNP is a non-interference index. BNP is unaffected by the administration of cardiovascular drugs and has no correlation with oxygenation index, alveolar-arterial oxygenation gradient, or urine volume. As a non-interference index in complicated clinical conditions, BNP may have a broader prospect in the application of PPHN.

BNP can be used in the diagnosis of PPHN. Previous studies have found that plasma BNP in infants with PPHN is significantly higher, as shown in Table 5 (97–100). Reynolds et al. (97) found that BNP could be used to differentiate PPHN from other respiratory diseases in patients with hypoxemia. Furthermore, Avitabile et al. (99) found that BNP had high specificity and positive predictive value in the diagnosis of PPHN and suggested that BNP could be used as a screening index for PPHN (Table 5).

**Table 5:** BNP in the diagnosis of PPHN.

**References**	***n***	**Age**	**BNP cutoff value (pg/ml)**	**Sensitivity (%)**	**Specificity (%)**
Reynolds et al. (97)	47	<24 h	550	83%	100%
Lewis (98)	60	<22 h	–	–	–
Avitabile et al. (99)	128	<24 h	130	50%	92%
Kandasamy (100)	25	–	117	94%	100%

BNP can be used to evaluate the severity of PPHN and to judge the prognosis and therapeutic effect. Reynolds et al. (97) found a strong correlation between the deterioration of clinical condition and increased BNP. Lewis (98) found that the initial BNP value of deceased patients with PPHN was significantly higher, suggesting that the initial BNP value can be used as an index to evaluate the prognosis of PPHN. Moreover, the BNP value decreased significantly in patients with effective treatment, suggesting that BNP can be used as an indicator of treatment effect evaluation and clinical follow-up. Behere et al. (101) also found that the probability of long-term complications and death was significantly higher in patients with higher initial BNP values, suggesting a poor prognosis. Furthermore, Fu and Zhang (96) found that the burst secretion of BNP may occur in the early stage of ventricular decompensation, which is the theoretical basis of BNP changes indicating PPHN deterioration. They also showed that the sensitivity, specificity, and accuracy were 88.97, 91.13, and 87.62%, respectively, in the prediction of poor prognosis by plasma BNP level.

Continuous measurement of the plasma BNP level is helpful to evaluate the clinical progress of PPHN and give corresponding management. Vijlbrief et al. (102) found that continuous monitoring of plasma BNP value during the cessation of treatment is helpful to predict recurrence. For recurrent patients, the plasma BNP value increased significantly after stopping treatment, and the change of BNP value was earlier than clinical manifestation. Therefore, they suggest to involve the BNP value as a predictor for stopping treatment in those PPHN infants. For infants with respiratory distress, hypoxemia, and BNP elevation, clinicians should be alert to the possibility of PPHN, make a clear diagnosis as soon as possible, and implement high-level nursing measures and supportive treatment. As the rapid rise of BNP in patients with PPHN may indicate poor curative effect or worsening of the disease, the treatment plan should be adjusted to perform timely ventilation intervention.

#### Application of BNP in Other Neonatal Respiratory Diseases

##### Application of BNP in Bronchopulmonary Dysplasia (BPD)

BPD is a respiratory disease that mainly affects premature infants and low birth weight infants and is related to prenatal lung injury or postpartum mechanical ventilation. Due to damage of the lung structure and function, patients with BPD are more prone to respiratory tract infection, chronic lung disease, and chronic pulmonary heart disease in the whole life process (103). The incidence of PH secondary to BPD (BPD-PH) is 18–43% (104), and the mortality rate at 2 years is up to 48% (101), representing a serious complication and an important cause of death. Screening and early diagnosis of infants with high risk of secondary pulmonary hypertension are conducive to early prevention, active monitoring and treatment, and improved prognosis.

The plasma BNP level can be used in the diagnosis, assessment, and prognosis evaluation of patients with BPD. Kalra et al. (104) found that the BNP level of premature infants with BPD was significantly higher, and that the plasma BNP concentration was positively correlated with the severity of disease. Infants with high BNP levels require longer mechanical ventilation and supportive treatment. They also highlighted that the increase in BNP may be related to the increase in pulmonary vascular pressure and the impairment of ventricular function; therefore, the determination of plasma BNP is helpful for risk stratification and treatment choice.

##### Application of BNP in CDH (Congenital Diaphragmatic Hernia)

CDH is a pediatric clinical emergency involving the respiratory and circulatory symptoms, respiratory failure caused by pulmonary hypoplasia and pulmonary hypertension is the main cause of death (105).

Partridge et al. (106) found that the plasma BNP concentration was significantly higher in patients with secondary pulmonary hypertension and that the BNP concentration gradually decreased to the normal level after effective surgery for CDH. They also suggested that BNP can be used to monitor the complications of CDH and to evaluate the therapeutic efficacy.

BNP can also be used to judge the prognosis of infants with CDH, as an assistant index of echocardiography. Steurer et al. (107) found that the plasma BNP concentration was significantly higher on the first day after birth in CDH infants with poor prognosis, while the accuracy of echocardiography in predicting the prognosis of CDH infants within 1 week after birth was poor. Moreover, Guslits et al. (108) found that for infants with CDH, a high BNP concentration 3–5 weeks after birth indicated that pulmonary hypertension had not been relieved, and the BNP concentration was an efficient index to judge the prognosis.

##### Application of BNP in Idiopathic Respiratory Distress Syndrome (IRDS)

IRDS is related to the deficiency of pulmonary surfactant and incomplete development of lung structure and has a higher incidence rate in preterm infants. Patients usually present with progressive dyspnea, intractable hypoxemia, and respiratory distress rapidly after birth. Severe cases can involve respiratory failure and multiple organ dysfunction and may eventually lead to death (109, 110).

For infants with respiratory distress, BNP is an important index to distinguish whether the symptoms come from respiratory or circulatory system. As mentioned above, infants with respiratory failure caused by circulatory diseases may be accompanied by a significant increase in plasma BNP, which does not occur in infants with only respiratory diseases. And as Koulouri et al. (111) suggested, plasma BNP concentration can be a good index to distinguish cardiogenic dyspnea from non-cardiogenic dyspnea, and it is helpful to select the appropriate treatment.

The BNP concentration can also be used to evaluate the clinical prognosis of patents with IRDS. Reel et al. (112) found that infants with severe IRDS are more likely to secondary pulmonary hypertension and leads to the increase of plasma BNP concentration. IRDS infants with elevated plasma BNP levels required a longer duration of mechanical ventilation, showed higher mortality and worse prognosis.

## Author Contributions

HX contributed to writing part of the review content and checking and summarizing the content. YH contributed to writing part of the review content and the layout and planning of the article. QC contributed to consulting, collecting and sorting out the early materials, and is responsible for writing part of the review. XH contributed to the concept of research, constructive discussion, and overall arrangement. All authors contributed to the article and approved the submitted version.

## Conflict of Interest

The authors declare that the research was conducted in the absence of any commercial or financial relationships that could be construed as a potential conflict of interest.

## Publisher's Note

All claims expressed in this article are solely those of the authors and do not necessarily represent those of their affiliated organizations, or those of the publisher, the editors and the reviewers. Any product that may be evaluated in this article, or claim that may be made by its manufacturer, is not guaranteed or endorsed by the publisher.
